# Moovis prosthesis for osteoarthritis of CMC joint of the thumb

**DOI:** 10.1186/1753-6561-9-S3-A90

**Published:** 2015-05-19

**Authors:** Pierre Jean Regnard

**Affiliations:** 1 SOS Mains Dijon, Point Medical, Dijon, 21000, France

## Introduction

The idea to create a new prosthesis was born in the year 2012 with the innovation ofa double mobility cup, as our orthopaedic colleagues were using for the hip. However, the challenge was to obtain the same mobility as the Elektra metal-on-metal prosthesis: 120° in all directions.

This new prosthesis is composed of 3 parts:

- An anatomic stem in titanium covered with Hydroxyapatite(HAP).

- A head covered by an insert of polyethylenewith 4 sizes of neck,

- A cup which is in 2 designs, press fit or screwed with a HAP coating.The diameter of the cup in titanium is 9.5mm.

The technique of implantation is almost the same as for the Elektra prosthesis.

3 important factors need to be adhered to.

○ Approach with care, especially with the branch of the dorsal sensory radial nerve, which is very close to the space between Abductor PollicisLongus and Extensor PollicisBrevis.

○ Freeing the base of the metacarpal is very importantin order to obtain a good view of the distal surface of the trapezium, because after an adequate resection of the base of the metacarpal and of the palmar beak it’s possible to dislocatethe metacarpalpalmarly.

○ To find the centre of the trapezium is easy, but extremely important, because of the size and final placement of the cup.

Our first implantation of the Moovis prosthesis was done in March 2013.

## Materials and methods

This is only a preliminary series because of very short follow-up. Mostly the indication was painful osteoarthritis of the CMC joint of the thumb, but in some cases, this prosthesis was used to change the cup after loosening of the Elektra’s or other prostheses’ cup. The advantage being it was possible to give a second chance toa previously implanted arthroplasty of the thumb, without using a cemented cup or performing a trapeziectomy.

**Figure 1:**
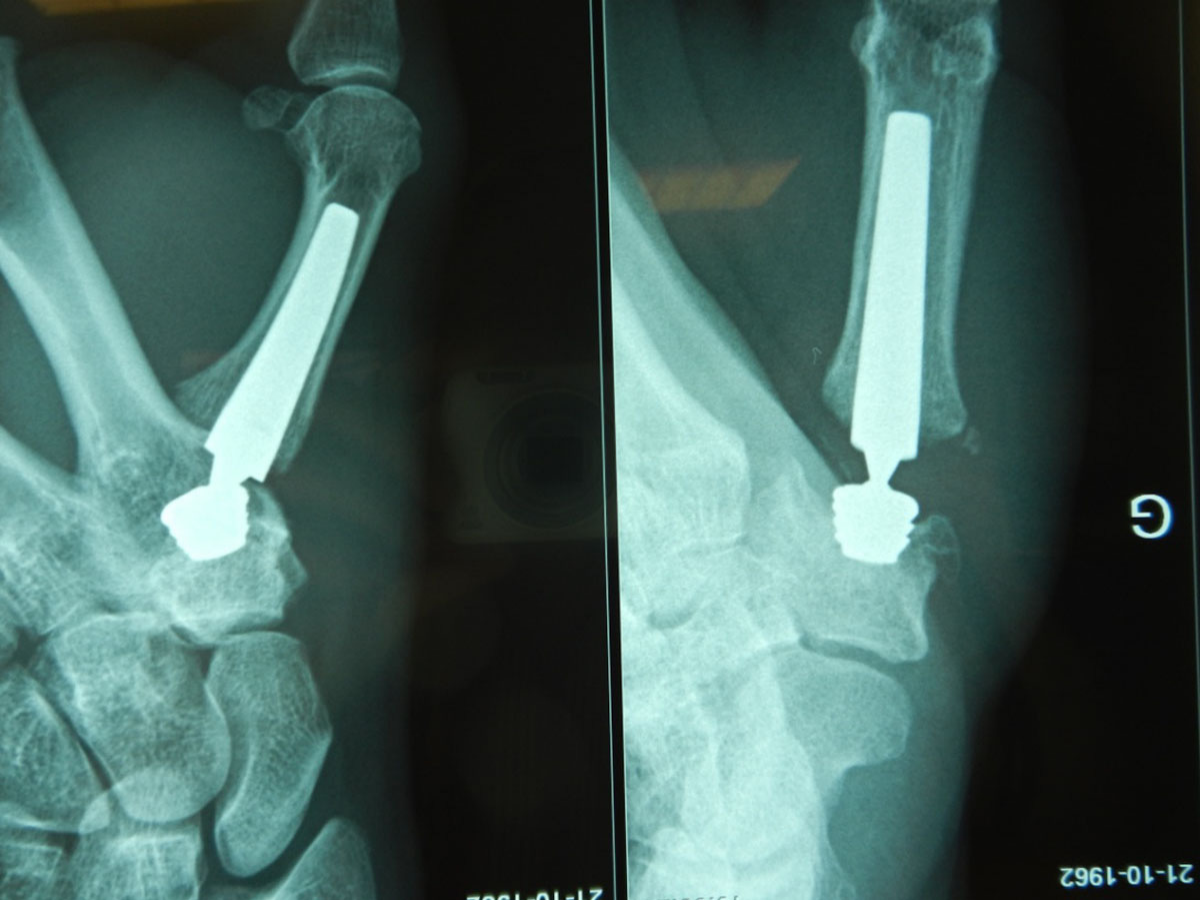
Moovis screwed into the Trapezium

**Figure 2:**
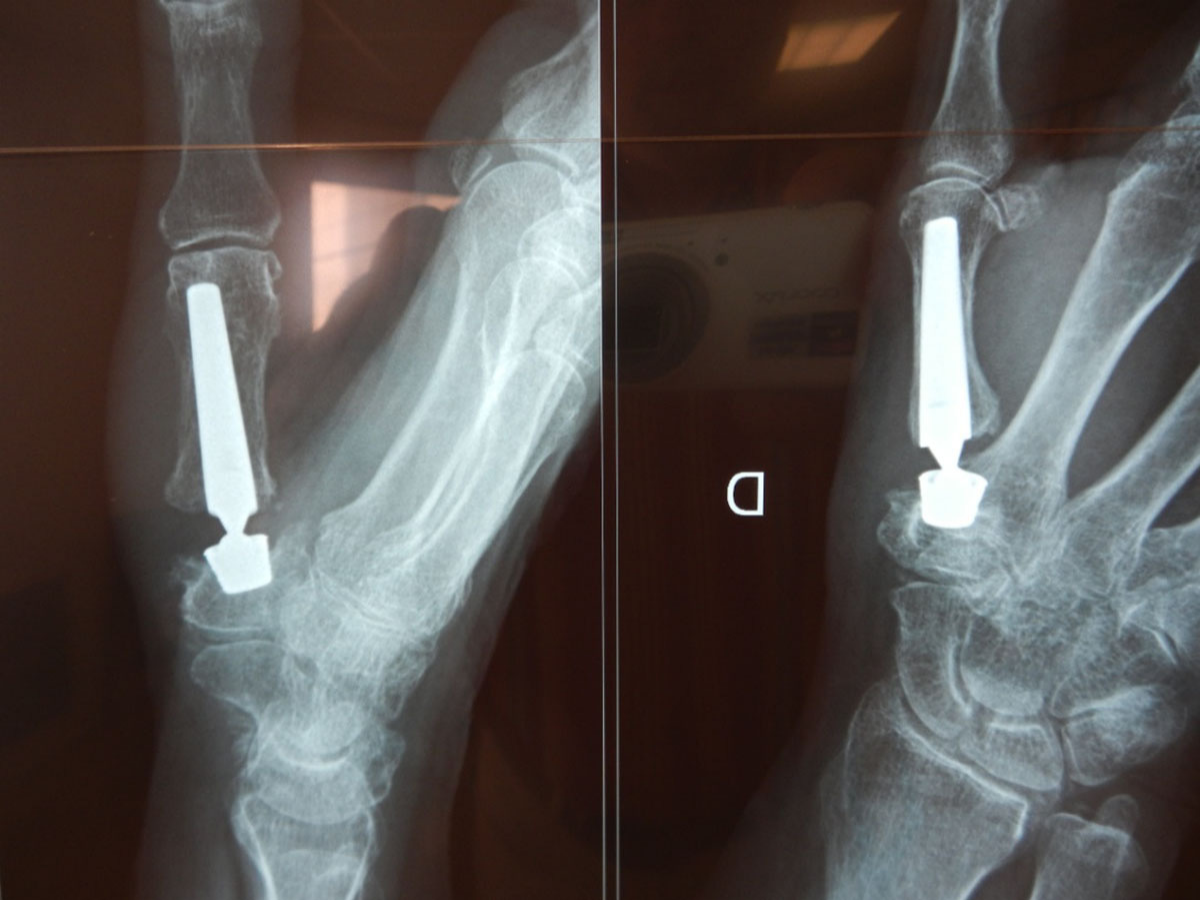
Moovis impacted into the Trapezium

In our series we have 57 cases of Moovis prosthesis with one case of bilateral implantation. Our patients were 38 females and 19 males. Twenty-three threaded cups (Fig 1) and thirty-four press-fit cups (Fig 2)were used, with fifty-one short and six mediumnecks.

## Results

For *mobility* the maximal angle between the first and the second metacarpal, the result was always between 40 and 45 °.The Kapandji’ scale for the opposition was between 8 and 10. Retropulsion with the palm laid flat on the table was always achievable.

The *strength* was measured with the Jamar for the global strength and compared with the other hand. For grip strength results were between 14 and 54 KgF, while for Key pinch the results were between 4 to 13 KgF. It was found that strength and key pinch really increasedonly after 3 months.

The *pain* disappeared in all cases but one, and that was the most important aspect for all the patients. This persistent pain was explained by osteoarthritis of the STT joint.

Time of recovery was rapid, after removing the splint which we applied for 3 weeks or 1 month, the function was almost normal, and only 3 patients needed rehabilitation.

The radiological aspect was almost always good, probably because of the size of the cup, the orientation was easy to achieve.

There were very few complications where only in one case it was impossible to obtain good fixation of the cup, because of an almost totally destroyed trapezium.

## Conclusion

Although the primary indication was osteoarthritis of CMC joint of the thumb at the 2^nd^, 3^rd^and 4^th^ stage of Dell’s classification, in some cases this prosthesis was very useful to repair loosening of the cup in other implanted prostheses, and thus to give a second chance to patients who have been operated many years before.

In this instance, the Moovis prosthesis avoids the need for implantation of a cemented cup, and/or trapeziectomy. Finally when STT osteoarthritis was present it was always possible to performan interposition arthroplasty for the STT, often with part of the APL simultaneously for this was still available.

In conclusion, although this series hasa very short follow-up, the results of the Moovis prosthesis were very good, without dislocation or loosening of the cup, and good recovery of function after a short duration.

